# Pseudoaneurysm of the uterine artery post loop electrosurgical excision procedure

**DOI:** 10.1186/s12905-021-01446-7

**Published:** 2021-08-16

**Authors:** Judith Ong, Kelvin Lee, Soe-Na Choo, Stanley Loh, Li Min Lim, Yee Yong Chen, Kuldip Singh

**Affiliations:** 1 Department of Obstetrics and Gynaecology, National University Hospital, Singapore, Singapore; 2 Department of Diagnostic Imaging, National University Hospital, Singapore, Singapore

**Keywords:** False aneurysm, Uterine artery, Uterine arterial embolisation, Cervical intraepithelial neoplasia, Angiography

## Abstract

**Background:**

The formation of a uterine artery pseudoaneurysm is rare and isolated cases have been reported in the existing literature following caesarean sections, curettages and cone biopsies. There has been no report of pseudoaneurysm formation following a loop electrosurgical excision procedure. Vaginal bleeding could potentially be life threatening if this diagnosis is not considered following cervical instrumentation or surgery. Management options range from haemostatic sutures, image-guided embolisation to surgical repair. We report the diagnosis and management of a case of uterine artery pseudoaneurysm after a loop electrosurgical excision procedure.

**Case presentation:**

A 37-year-old woman was diagnosed with cervical intraepithelial neoplasia grade 3 (CIN3) and underwent a therapeutic loop electrosurgical excision procedure. One month after the procedure, the patient presented to the emergency department with repeated episodes of sudden-onset heavy vaginal bleeding associated with hypotension and syncope. A computed tomography angiogram was performed, which demonstrated a pseudoaneurysm of the right uterine artery. Following the diagnosis, image-guided embolisation was performed successfully. Post-embolisation angiograms showed successful embolisation of the pseudoaneurysm and the patient had no further episodes of bleeding.

**Conclusions:**

Loop electrosurgical excision procedures are generally safe but rarely, can be complicated by the formation of uterine artery pseudoaneurysms. The depth of the loop electrosurgical excision procedure and vascular anatomy should be considered to prevent such complications. A computed tomography angiogram appears to be ideal for diagnosis. Image-guided embolisation is safe and effective as a therapeutic measure, with minimal morbidity.

## Background

Loop electrosurgical excision procedure (LEEP) is a procedure typically used for the treatment of cervical intraepithelial neoplasia (CIN). These procedures are commonly performed and are generally considered as minor procedures with minimal complications. Light bleeding is common post procedure, but life-threatening bleeding is a rare occurrence [1]. There have been isolated case reports of heavy bleeding arising from a uterine artery pseudoaneurysm (UAP) formation after cervical conization, caesarean sections and curettages but none following LEEP procedures, to our knowledge.

In existing literature, the diagnosis of a UAP is confirmed with imaging techniques such as ultrasound and computed tomography (CT) angiography [2–5]. These imaging methods may be performed in isolation or in combination. Management techniques vary from haemostatic sutures, surgical vascular repair to CT-guided embolisation [2–6].

In this report, the diagnosis and management of a uterine artery pseudoaneurysm causing severe vaginal bleeding after a loop electrosurgical excision procedure is presented.

## Case presentation

A 37-year-old woman, para 4 presented to the emergency department 3 times over 20 days for sudden onset of heavy vaginal bleeding after a LEEP procedure. The patient underwent a LEEP for CIN3 in a nearby local hospital. There was slightly more bleeding than expected during the procedure, however routine haemostatic measures sufficed. The LEEP specimen was 22 mm in length × 17 mm in diameter. In the days following the procedure, recovery was uneventful with only vaginal spotting.

The patient first presented to the emergency department one month after the LEEP procedure with a sudden onset of heavy bleeding and syncope. Her blood pressure was 88/45 mmHg and heart rate 80 beats/min on arrival. The abdomen was soft and non-tender. Speculum examination revealed no active bleeding with an expected appearance of the cervix post LEEP. The patient’s haemoglobin level was 9.2 g/dL and was transfused one pack of red blood cells (RBCs). Her urine pregnancy test was negative and coagulation results were also unremarkable. As her bleeding had ceased, she was discharged from the hospital 2 days later with a haemoglobin of 12 g/dL. The patient was commenced on tranexamic acid.

The patient then represented one week later with another sudden onset of heavy bleeding and hypotension. Her blood pressure was 75/40 mmHg and heart rate 80 beats/min. Similarly, on examination, her abdomen was soft, speculum revealed no further active bleeding with a normal cervix which was fully epithelialised. Her haemoglobin was 7.5 g/dL and coagulation screen was normal. The patient was transfused 2 packs of RBCs with an initial diagnosis of abnormal uterine bleeding and was counseled on the need for further work-up in the outpatient clinic. She was given one dose of intramuscular progesterone, then continued on oral norethisterone and tranexamic acid on discharge.

Four days later, she returned the third time to the emergency department with another episode of sudden onset of massive bleeding per vaginum with hypotension and syncope. The patient’s blood pressure on arrival was 80/60 mmHg and heart rate was 100 beats/min. Her haemoglobin was 7.2 g/dL and transfusion of RBCs was commenced once more. Examination was unremarkable with no further bleeding once the patient arrived.

**Fig. 1:**
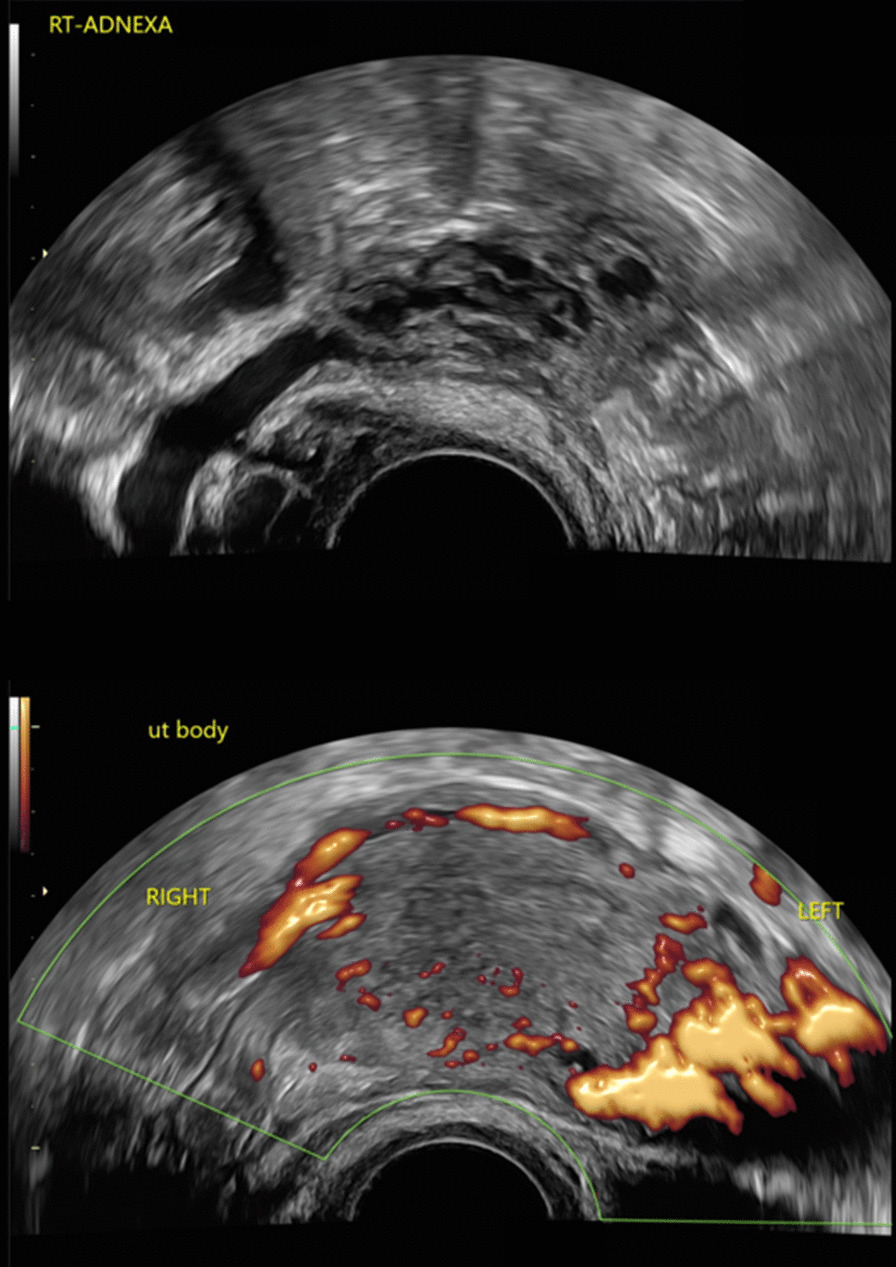
Transvaginal ultrasound images: Colour flow mapping demonstrated tortuous dilated vasculatures over the cervix, appearing more prominent over the paracervical region

**Fig. 2:**
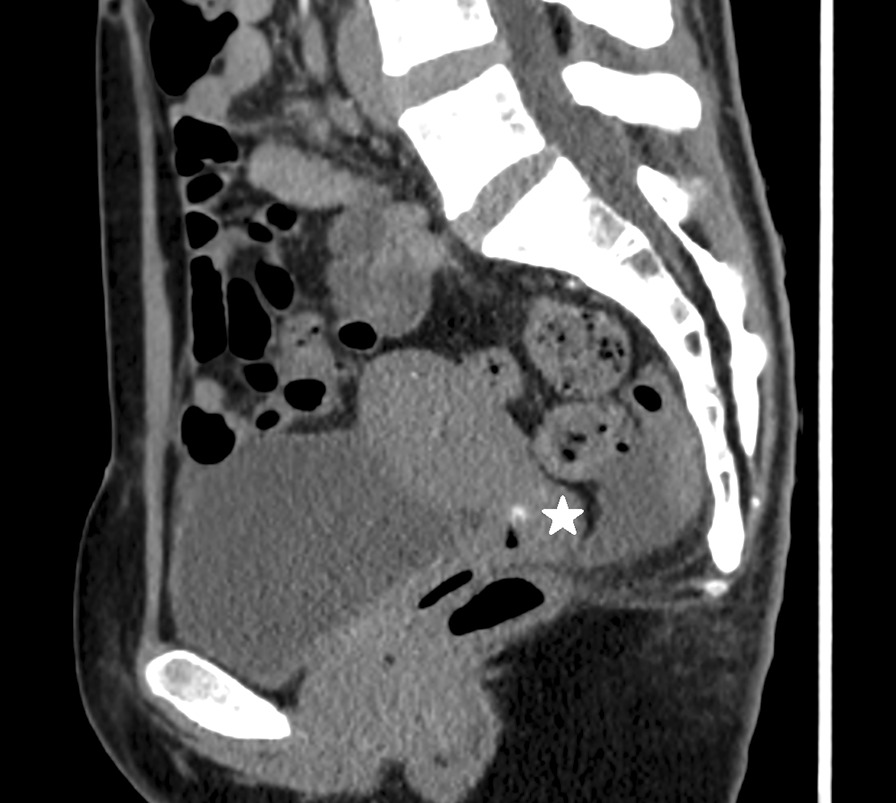
CT Angiogram prior to embolisation: Sagittal reconstruction of the pelvis on CT angiogram shows a small pseudoaneurysm (white star) of the uterine artery located at the cervical–vaginal junction

**Fig. 3:**
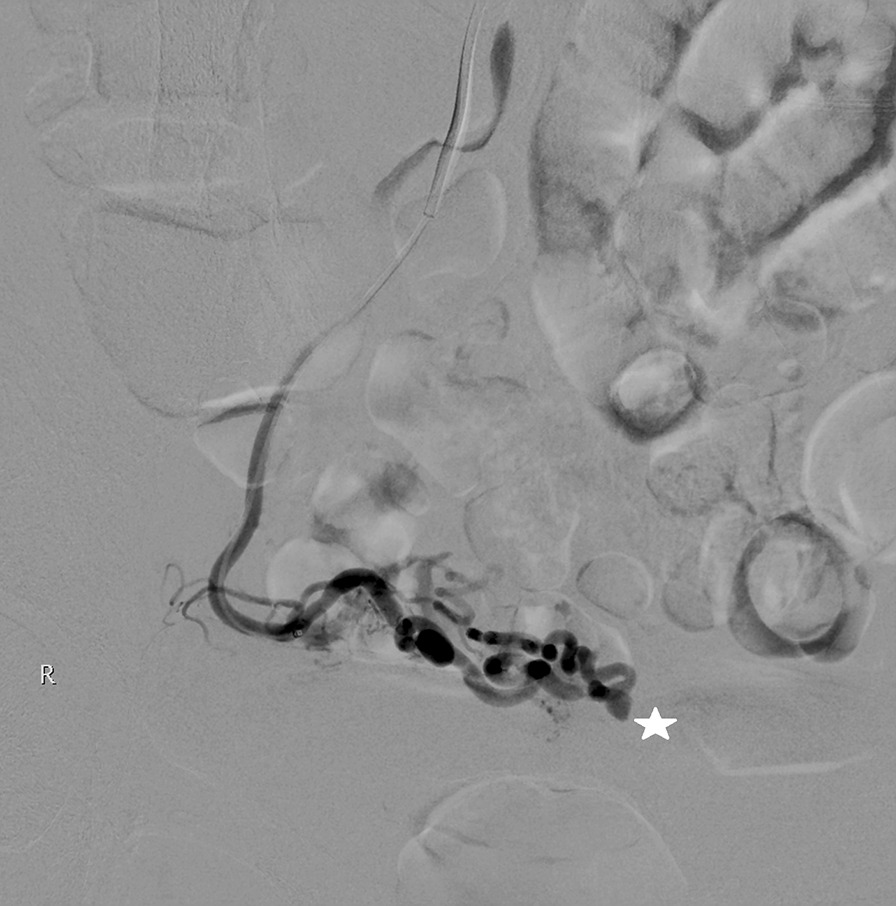
Angiogram prior to embolisation: Angiogram was performed following cannulation of the right uterine artery showing a pseudoaneurysm corresponding to the finding on the CT angiogram

In view of the repeated episodes of sudden onset of heavy vaginal bleeding, a vascular malformation was suspected. A pelvic ultrasound was initially performed which showed a normal uterus with a thin endometrium of 3.3 mm. Ovaries were normal and torturous dilated vasculature were seen over the cervix, appearing more prominent over the paracervical region. Decision was made for a CT Angiogram as the pelvic ultrasound was non diagnostic. A CT Angiogram was then performed which revealed a 0.5 cm focal arterial hyperdensity at the utero-cervical junction which was suspicious for a pseudoaneurysm (Fig. 1). The patient then underwent a transcatheter angiogram which confirmed the presence of the right uterine artery pseudoaneurysm. It was not bleeding on initial angiograph. However, during the angiogram there was inadvertent rupture of the pseudoaneurysm with gentle passage of a microguidewire, confirming the diagnosis that this was the cause of repeated bleeding (Figs. 2, 3). Embolisation was then performed using a combination of Gelfoam® slurry and also a 0.3 cc of a liquid embolic Phil™ 25. Post-embolisation angiograms showed successful embolization of the pseudoaneurysm (Fig. 3). The left internal iliac and the left uterine artery were also cannulated and angiograms were performed, demonstrating no supply to the pseudoaneurysm. The patient tolerated the procedure well with minimal pain. She was observed inpatient for complications and subsequently discharged from the hospital two days after the embolization without incidents. There were no further episodes of vaginal bleeding thereafter.

**Fig. 4:**
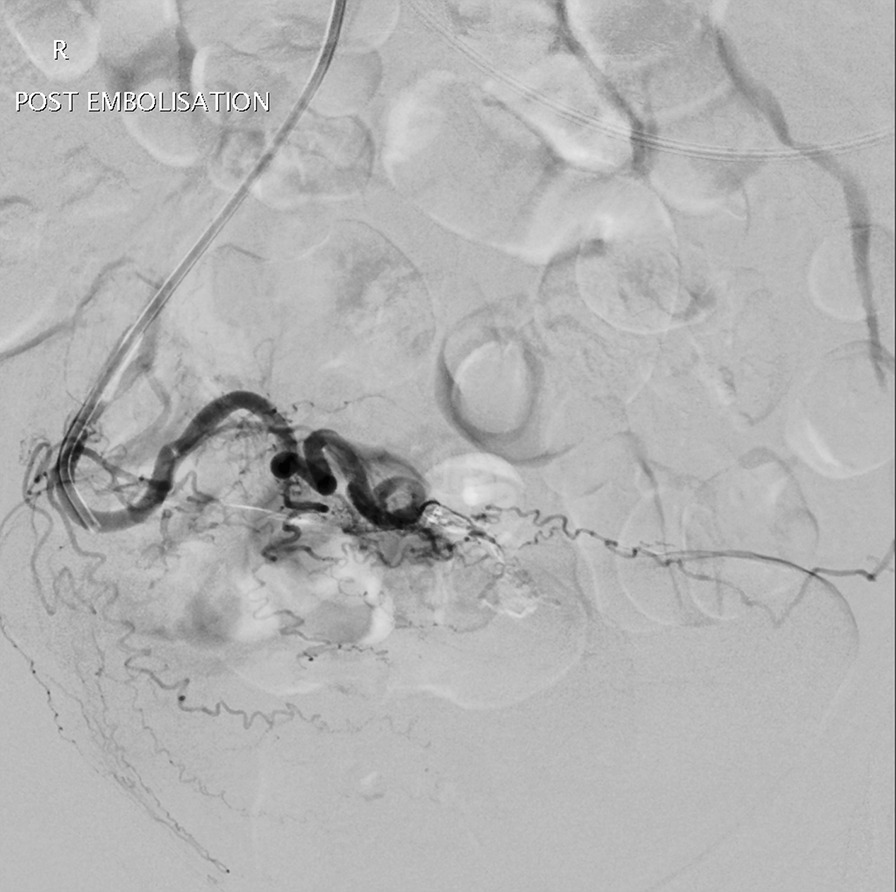
Angiogram post embolization: The Pseudoaneurysm has been successfully embolised with no further contrast extravasation

A follow-up CT angiogram was also performed a month later which showed successful treatment of the pseudoaneurysm with no evidence of recurrence (Fig. 4).

## Discussion and conclusions

Pseudoaneurysms or false aneurysms form as a result of vascular injury [2]. It is defined an abnormal outpouching of an artery, where blood collects extra luminally and is only surrounded by the outermost layer of the arterial wall. In the event an artery is injured and not adequately repaired, blood escapes and dissects through the arterial wall forming a pseudoaneurysm. As this is a weakened area, once formed, it can easily rupture causing profuse and life threatening bleeding [3].

The formation of a UAP is rare. In existing literature there are isolated case reports of UAP formation. In obstetrics, they have been reported post Caesarean sections and vaginal deliveries [3–6]. In gynaecology, isolated reports of pseudoaneurysm post curettage and conization of the cervix exist [2, 7–9], however, there has been no reports following LEEP procedures. One of the possible reasons behind this could be that LEEP procedures are typically more superficial and less invasive in comparison to conization of the cervix or curettages. The cervix of a woman of reproductive age approximately measures 35 mm in length and 20 mm in diameter [10, 11]. In existing literature, CIN has been shown to penetrate the endocervical stroma mostly at a depth of 3–3.5 mm, occasionally to a depth of 6 mm [10, 11]. Thus, the suggested depth of excision would be at a maximum of 8 mm [10, 11]. It is known with increasing depth of excision, risks of complications such as bleeding, stenosis and preterm birth increase. It is also important to be aware of the vascular anatomy of the cervix. The descending branches of the uterine artery and vein which supply the cervix enter at the 3 and 9 o’clock positions [12]. Care should be taken during vasopressin infiltration in these regions but also the excision of the cervix too laterally. In this case, the specimen was 22 mm in length and 17 mm in diameter, its depth and size possibly contributing to this vascular complication.

Majority of the UAP cases were managed successfully with first-line image guided embolization. In several cases where the initial cause was uncertain, other methods such as dilatation and curettage or bakri balloon insertion were attempted. However, as these did not cease the bleeding, image guided emergency embolisation was eventually performed [5]. In one report where embolization was not performed, the patient required an exploratory laparotomy for diagnosis and therapeutic measures [4].

CT Angiogram is indeed an important diagnostic tool and has been used successfully here and in previous cases [2, 6–8]. This would be expected to show an irregular outpouching from the uterine artery.

Embolisation of the pseudoaneurysm as a therapeutic measure appears to be effective, safe, and minimally invasive [2, 6–8]. In both this case and existing literature, when embolisation was performed, there have been no reported morbidities and patients had not returned with recurrences. Other methods include surgical methods such as haemostatic sutures, vascular repair and hysterectomy as a last resort [4]. However, these are more invasive, with longer recovery time and could potentially result in further morbidity.

In conclusion, UAP can occur as a rare complication with any cervical procedure, including an endometrial curettage, a LEEP or conization of the cervix. The depth of the LEEP and vascular anatomy should be considered to prevent such complications. The diagnosis of a vascular malformation should be considered following cervical surgery if there is a sudden onset of brisk vaginal bleeding. CT Angiogram should be considered for the diagnosis of a pseudoaneurysm and targeted embolisation as a non-invasive therapeutic measure.

## Data Availability

The images analysed are available from the corresponding author on reasonable request.

## Abbreviations

LEEP: Loop electrosurgical excision procedure
CIN: Cervical intraepithelial neoplasia
UAP: Uterine artery pseudoaneurysm
CT: Computed tomography
RBCs: Red blood cells
